# Supplementary material for: Identification of Cyclic-di-GMP-Modulating Protein Residues by Bidirectionally Evolving a Social Behavior in Pseudomonas fluorescens
Source: mSystems. 2022 Oct 3;7(5):e00737-22. doi: 10.1128/msystems.00737-22 (PMC9600634; doi:10.1128/msystems.00737-22)
Supplement: TABLE S1 [file msystems.00737-22-s0004.docx]

| **Supplemental Table S1.** | | | | | | | | | | | |
| --- | --- | --- | --- | --- | --- | --- | --- | --- | --- | --- | --- |
| Isolate ID § | Generation §§ | Motility Assay † | LC-MS/MS (nM/OD 600) ‡ | | | Locus *** | CDS ID *** | Mutation Location (NT) *** | Consequence *** | Genotype (AA) *** | Genotype (NT) *** |
|  |  |  | Replicate A | Replicate B | Replicate C |  |  |  |  |  |  |
| WT # | WT | motile | 59.23 | 73.16 | 62.21 | na | na | na | na | na | na |
| M * | M0 | motile | 49.19 | 51.54 | 48.64 | RsmE | Pfl01_1912 ← | 2186046 | Frame shift | Frame shift | ∆1 bp 126/195 nt |
| D ** | D0 | non-motile | 187.52 | 146.52 | 156.37 | WspC | Pfl01_1054 → | 1222594 | In-frame readthrough | In-frame readthrough | ∆2 bp 1269-1270/1278 nt |
| 1.0 ** | M0 | motile | 49.64 | 58.15 | 53.51 | WspC | Pfl01_1054 → | 1221330 | In-frame ∆ | ∆*wspC* | ∆5‑1278/1278 nt |
| 1.1 | D0 | non-motile | 80.66 | 94.75 | 112.01 | WspA | Pfl01_1052 → | 1220312 | Missense | A381V | GCC→GTC |
| 1.2 | M1 | motile | 52.28 | 59.70 | 47.26 | WspB | Pfl01_1053 → | 1221196 | Missense | L135P | CTG→CCG |
| 1.3a | D1 | non-motile | 63.54 | 81.09 | 71.31 | WspE | Pfl01_1056 → | 1225308 | In-frame ∆ | ∆674-696 | ∆2029-2097/2295 nt |
| 1.3b | D1 | non-motile |  |  |  | WspE | Pfl01_1056 → | 1225307 | Duplication | 673GMD 1→2 | +9bp 2028/2295 nt |
| 1.4 | M2 | motile | 56.99 | 49.71 | 49.42 | WspE | Pfl01_1056 → | 1224117 | Frame shift | 289* | +1bp 838/2295 |
| 1.5a | D2 | non-motile | 290.55 | 328.67 | 307.72 | DgcH | Pfl01_0050 ← | 55870 | Missense | V459G | GTC→GGC |
| 1.5b | D2 | non-motile |  |  |  | DgcH | Pfl01_0050 ← | 55317 | Duplication | +472-643 @ 643 | +513bp 1929/2091 nt |
| 1.5c | D2 | non-motile |  |  |  | Pfl01_3026 | Pfl01_3026 → | 3463653 | Missense | T242I | ACC→ATC |
| 2.1 | D0 | non-motile | 167.26 | 267.13 | 209.38 | WspA | Pfl01_1052 → | 1220020 | In-frame ∆ | ∆283-310 | ∆850‑933/1623 nt |
| 2.2 | M1 | motile | 47.27 | 41.65 | 36.89 | WspD | Pfl01_1055 → | 1222868 | Missense | W90L | TGG→TTG |
| 2.3 | D1 | non-motile | 77.37 | 81.28 | 110.54 | NarA | Pfl01_3147 → | 3613968 | Missense | A383S | GCC→TCC |
| 2.4 | M2 | motile |  |  |  | WspD | Pfl01_1055 → | 1223197 | Nonsense | R200* | CGA→TGA |
| 2.5 | D2 | non-motile |  |  |  | GcbC | Pfl01_4665 →/ → Pfl01_4666 | 5266677 | 5' UTR | + Pfl01_2031-2032 | +1316 +54/‑56 |
| 2.6 | M3 | motile |  |  |  | WspA | Pfl01_1052 → | 1219276 | Nonsense | E36* | GAA→TAA |
| 2.7 | D3 | non-motile |  |  |  | DgcH | Pfl01_0050 ← | 55909 | In-frame ∆ | ∆441-446 | ∆15bp 1323-1337/2091 nt |
| 3.1 | D0 | non-motile | 349.34 | 359.63 | 363.90 | WspA | Pfl01_1052 → | 1220059 | In-frame ∆ | ∆297-310 | ∆889‑930/1623 nt |
| 3.2 | M1 | motile | 49.45 | 51.62 | 51.41 | WspA | Pfl01_1052 → | 1220330 | Missense | L387R | CTG→CGG |
| 3.3a | D1 | non-motile | 127.36 | 112.64 | 160.92 | WspE | Pfl01_1056 → | 1225081 | Missense | G601V | GGC→GTC |
| 3.3b | D1 | non-motile |  |  |  | WspE | Pfl01_1056 → | 1224300 | Missense | E341K | GAA→AAA |
| 3.4 | M2 | motile |  |  |  | WspR | Pfl01_1058 → | 1227186 | Missense | L186Q | CTG→CAG |
| 3.5 | D2 | non-motile |  |  |  | YfiN | Pfl01_0692 → | 818804 | Missense | G157A | GGT→GCT |
| 3.6 | M3 | motile |  |  |  | YfiN | Pfl01_0692 → | 819473 | In-frame ∆ | ∆381-385 | ∆1139-1153/1269 nt |
| 3.7a | D3 | non-motile |  |  |  | DgcX | Pfl01_3550 → | 4023270 | Missense | M350I | ATG→ATA |
| 3.7b | D3 | non-motile |  |  |  | GcbC | Pfl01_4665 →/ → Pfl01_4666 | 5266677 | 5' UTR | + Pfl01_2031-2032 | +1316 +54/‑56 |
| 3.7c | D3 | non-motile |  |  |  | MorA | Pfl01_4876 → | 5499015 | Missense | M991I | ATG→ATA |
| 3.7d | D3 | non-motile |  |  |  | MorA | Pfl01_4876 → | 5499101 | Missense | A1020E | GCG→GAG |
| 4.1 | D0 | non-motile | 145.66 | 170.85 | 148.76 | DgcH | Pfl01_0050 ← | 55647 | In-frame ∆ | ∆524-537 | ∆1558‑1599/2091 nt |
| 4.2 | M1 | motile | 42.78 | 46.36 | 47.13 | DgcH | Pfl01_0050 ← | 55345 | Missense | L634P | CTC→CCC |
| 4.3a | D1 | non-motile | 392.53 | 328.69 | 404.68 | WspA | Pfl01_1052 → | 1220309 | Missense | V380A | GTC→GCC |
| 4.3b | D1 | non-motile |  |  |  | WspF | Pfl01_1057 → | 1226046 | Missense | S159L | TCG→TTG |
| 4.3c | D1 | non-motile |  |  |  | WspA | Pfl01_1052 → | 1220170 | In-frame ∆ | ∆334-336 | ∆1000-1008/1623 nt |
| 4.4 | M2 | motile | 50.14 | 53.49 | 61.31 | WspE | Pfl01_1056 → | 1224447 | Missense | D390N | GAC→AAC |
| 4.5a | D2 | non-motile | 81.95 | 81.25 | 75.76 | WspA | Pfl01_1052 → | 1220020 | In-frame ∆ | ∆283-310 | ∆850‑933/1623 nt |
| 4.5b | D2 | non-motile |  |  |  | WspA | Pfl01_1052 → | 1219961 | Missense | Q264L | CAG→CTG |
| 4.6a | M3 | motile | 57.50 | 54.72 | 51.88 | WspE | Pfl01_1056 → | 1224601 | Missense | V441A | GTG→GCG |
| 4.6b | M3 | motile | 54.52 | 56.68 | 55.08 | WspD | Pfl01_1055 → | 1223058 | In-frame ∆ | ∆152-157 | ∆459-476/684 nt |
| 4.7a | D3 | non-motile | 275.45 | 268.03 | 177.32 | WspE | Pfl01_1056 → | 1224693 | Missense | S472C | AGT→TGT |
| 4.7b | D3 | non-motile |  |  |  | GcbC | Pfl01_4665 →/ → Pfl01_4666 | 5266677 | 5' UTR | + Pfl01_2031-2032 | +1316 +54/‑56 |
| 4.7c | D3 | non-motile |  |  |  | FleN | Pfl01_1562 → | 1752356 | Missense | T13I | ACC→ATC |
| 4.7d | D3 | non-motile |  |  |  | CysH | Pfl01_1757 ← | 1952544 | Nonsense | W51* | TGG→TGA |
| 4.8 | M4 | motile |  |  |  | GcbC | Pfl01_4666 → | 5268046 | Frame shift | Frame shift | +1bp 1314/1506 nt |
| 4.9a | D4 | non-motile |  |  |  | DgcY | Pfl01_3502 | 3982758 | Frame shift | Frame shift | ∆502-555/672 nt |
| 4.9b | D4 | non-motile |  |  |  | MorA | Pfl01_4876 → | 5498817 | Missense | M925I | ATG→ATC |
| 5.1 | D0 | non-motile | 106.41 | 95.85 | 119.21 | WspF | Pfl01_1057 → | 1225883 | Missense | P105S | CCG→TCG |
| 5.2 | M1 | motile | 39.11 | 39.97 | 57.53 | WspA | Pfl01_1052 → | 1220156 | In-frame ∆ | ∆329 | ∆986‑988/1623 nt |
| 5.3a | D1 | non-motile | 158.40 | 145.92 | 166.92 | WspA | Pfl01_1052 → | 1220017 | In-frame ∆ | ∆283-296 | ∆847-888/1623 nt |
| 5.3b | D1 | non-motile |  |  |  | WspA | Pfl01_1052 → | 1220020 | In-frame ∆ | ∆283-310 | ∆850‑933/1623 nt |
| 5.4 | M2 | motile | 40.20 | 67.63 | 44.65 | WspD | Pfl01_1055 → | 1223058 | In-frame ∆ | ∆152-157 | ∆459-476/684 nt |
| 5.5a | D2 | non-motile | 131.86 | 92.83 | 93.92 | SadC | Pfl01_4451 ← | 5013023 | 5' UTR | 5' UTR | A→T (-11) |
| 5.5b | D2 | non-motile |  |  |  | SadC | Pfl01_4451 ← | 5013023 | 5' UTR | 5' UTR | A→C (-11) |
| 5.5c | D2 | non-motile |  |  |  | SadC | Pfl01_4451 ← | 5013023 | 5' UTR | 5' UTR | A→C (-11) |
| 5.5d | D2 | non-motile |  |  |  | WspE | Pfl01_1056 → | 1224211 | Duplication | 308RMV 1→2 | +9bp 932/2295 |
| 5.6a | M3 | motile | 49.79 | 50.94 | 43.86 | SadC | Pfl01_4451 ← | 5012937 | In-frame ∆ | 25L 4→3 | ∆74‑76/1107 nt |
| 5.6b | M3 | motile |  |  |  | SadC | Pfl01_4451 ← | 5012937 | In-frame ∆ | 25L 4→3 | ∆74‑76/1107 nt |
| 5.7a | D3 | non-motile | 68.79 | 59.84 | 78.79 | IlvH | Pfl01_4787 ← | 5402643 | Missense | S149R | AGT→AGG |
| 5.7b | D3 | non-motile |  |  |  | DgcH | Pfl01_0050 ← | 55647 | In-frame ∆ | ∆524-537 | ∆1558‑1599/2091 nt |
| 5.7c | D3 | non-motile |  |  |  | WspE | Pfl01_1056 → | 1225351 | Missense | D691G | GAC→GGC |
| 5.7d | D3 | non-motile |  |  |  | WspE | Pfl01_1056 → | 1224324 | Missense | R349S | CGC→AGC |
| 5.7e | D3 | non-motile |  |  |  | YfiN | Pfl01_0692 → | 818804 | Missense | G157A | GGT→GCT |
| 5.8a | M4 | motile | 54.44 | 51.70 | 73.94 | ParA | Pfl01_0730 → | 858438 | Missense | D115N | GAT→AAT |
| 5.8b | M4 | motile | 47.61 | 37.67 | 40.41 | YfiN | Pfl01_0692 → | 818969 | Missense | I212T | ATT→ACT |
| 5.9a | D4 | non-motile | 329.69 | 230.96 | 263.57 | WspA | Pfl01_1052 → | 1220649 | In-frame ∆ | wspA::D | ∆2421 bp |
| 5.9b | D4 | non-motile | 92.77 | 95.21 | 136.41 | WspB | Pfl01_1053 → | 1220923 | In-frame ∆ | wspB::D | ∆1840 bp |
| 5.9c | D4 | non-motile |  |  |  | WspE | Pfl01_1056 → | 1223451 | Missense | R58W | CGG→TGG |
| 5.9d | D4 | non-motile |  |  |  | WspE | Pfl01_1056 → | 1223648 | Missense | M123I | ATG→ATA |
| 5.9e | D4 | non-motile | 53.36 | 63.66 | 60.18 | IlvH | Pfl01_4787 ← | 5402642 | Missense | G150S | GGC→AGC |
| 5.10a | M5 | motile | 56.96 | 56.62 | 55.12 | IlvI | Pfl01_4788 ← | 5403629 | Frame shift | Frame shift | ∆23bp 1166-1188/1725 nt |
| 5.10b | M5 | motile | 49.96 | 49.89 | 107.61 | IlvI | Pfl01_4788 ← | 5403629 | Frame shift | Frame shift | ∆17bp 1172-1188/1725 nt |
| 5.10c | M5 | motile |  |  |  | WspR | Pfl01_1058 → | 1227150 | Missense | T174I | ACC→ATC |
| 5.11a | D5 | non-motile | 110.47 | 109.78 | 180.81 | WspE | Pfl01_1056 → | 1223648 | Missense | M123I | ATG→ATA |
| 5.11b | D5 | non-motile | 104.23 | 129.78 | 152.89 | WspE | Pfl01_1056 → | 1225239 | In-frame ∆ | ∆653-656 | ∆12bp 1960-1971/2295 nt |
| 5.11c | D5 | non-motile | 81.07 | 77.14 | 96.70 | WspE | Pfl01_1056 → | 1224144 | Missense | D289N | GAC→AAC |
| 5.11d | D5 | non-motile | 128.07 | 115.76 | 132.55 | DgcH | Pfl01_0050 ← | 55647 | In-frame ∆ | ∆524-537 | ∆1558‑1599/2091 nt |
| 5.11e | D5 | non-motile |  |  |  | GcbC | Pfl01_4665 →/ → Pfl01_4666 | 5266677 | 5' UTR | + Pfl01_2031-2032 | +1316 +54/‑56 |
| 5.11f | D5 | non-motile |  |  |  | MorA | Pfl01_4876 → | 5499284 | Missense | E1081A | GAA→GCA |
| 5.12 | M6 | motile |  |  |  | GcbC | Pfl01_4666 → | 5266862 | Missense | A44P | GCC→CCC |
| 5.13a | D6 | non-motile |  |  |  | MorA | Pfl01_4876 → | 5499088 | In-frame ∆ | ∆1015-1020 | ∆15bp 3046-3060/3849 nt |
| 5.13b | D6 | non-motile |  |  |  | GcbC | Pfl01_4666 → | 5267723 | Missense | A331T | GCC→ACC |
| 6.1 | D0 | non-motile |  |  |  | WspF | Pfl01_1057 → | 1226544 | Frame shift | KI325R* | ∆974/1011 nt |
| 6.2a | M1 | motile |  |  |  | WspA | Pfl01_1052 → | 1219239 | Duplication | L23 2→3 | +3bp 69/1623 |
| 6.2b | M1 | motile |  |  |  | WspR | Pfl01_1058 → | 1227374 | Missense | G249C | GGC→TGC |
| 6.3 | D1 | non-motile |  |  |  | WspA | Pfl01_1052 → | 1219239 | Duplication | L24 3→2 | -3bp 69/1623 |
| 6.4 | M2 | motile | 51.19 | 81.77 | 61.87 | WspE | Pfl01_1056 → | 1225435 | Frame shift | 748* | +1bp 2156/2295 nt |
| 6.5a | D2 | non-motile | 189.04 | 141.94 | 135.43 | DgcY | [Pfl01_3505]–Pfl01_3506 | 3985701 | In-frame ∆ | 124* ∆5' UTR 3508 | ∆3505,∆3506,∆3508 5'UTR |
| 6.5b | D2 | non-motile |  |  |  | SadC | Pfl01_4451 ← | 5013023 | 5' UTR | 5' UTR | A→C (-11) |
| 6.6a | M3 | motile | 69.41 | 59.49 | 94.44 | DgcY | [Pfl01_3500]–Pfl01_3510 | 3981080 | In-frame ∆ | ∆10 proteins | ∆3500-3510 |
| 6.6b | M3 | motile |  |  |  | SadC | Pfl01_4451 ← | 5012937 | In-frame ∆ | 25L 4→3 | ∆74‑76/1107 nt |
| 6.7a | D3 | non-motile |  |  |  | CheB | Pfl01_1567 | 1758645 | Frame shift | Frame shift | ∆1bp 1061/1137 nt |
| 6.7b | D3 | non-motile |  |  |  | FlgF | Pfl01_1501 → | 1680303 | Frame shift | Frame shift | ∆14bp 138-151/741 nt |
| 6.7c | D3 | non-motile | 66.67 | 50.69 | 60.25 | YfiN | Pfl01_0692 → | 818803 | Missense | G157C | GGT→TGT |
| 6.7d | D3 | non-motile |  |  |  | DgcH | Pfl01_0050 ← | 55952 | Frame shift | ∆430 | ∆1292-1294/2091 nt |
| 6.7e | D3 | non-motile |  |  |  | YfiR | Pfl01_0691 → | 817876 | In-frame ∆ | ∆37-61 | ∆114-188/576 nt |
| 6.8a | M4 | motile | 41.34 | 42.22 | 39.91 | CdrB | Pfl01_0652 ← | 763630 | Missense | A290T | GCC→ACC |
| 6.8b | M4 | motile |  |  |  | YfiN | Pfl01_0692 → | 819458 | Missense | A375V | GCG→GTG |
| 6.8c | M4 | motile |  |  |  | YfiN | Pfl01_0692 → | 818709 | Frame shift | Frame shift | +1bp 375/1269 nt |
| 6.9a | D4 | non-motile |  |  |  | DgcH | Pfl01_0050 ← | 55978 | Missense | L423P | CTC→CCC |
| 6.9b | D4 | non-motile |  |  |  | MorA | Pfl01_4876 → | 5499015 | Missense | M991I | ATG→ATA |
| 6.9c | D4 | non-motile |  |  |  | MorA | Pfl01_4876 → | 5499015 | Missense | M991I | ATG→ATT |
| 6.9d | D4 | non-motile |  |  |  | DgcH | Pfl01_0050 ← | 55647 | In-frame ∆ | ∆524-537 | ∆1558‑1599/2091 nt |
| 6.9e | D4 | non-motile |  |  |  | GcbC | Pfl01_4665 →/ → Pfl01_4666 | 5266677 | 5' UTR | + Pfl01_2031-2032 | +1316 +54/‑56 |
| 6.9f | D4 | non-motile |  |  |  | RapA | Pfl01_1678 ← | 1873535 | Missense | S317A | TCG→GCG |
| 7.1 | D0 | non-motile | 187.52 | 146.52 | 156.37 | WspC | Pfl01_1054 → | 1222594 | In-frame ∆ | *wspC::D* | ∆1269‑1270/1278 nt |
| 7.2a | M1 | motile |  |  |  | WspE | Pfl01_1056 → | 1223360 | Frame shift | 156* | +1bp 81/2295 nt |
| 7.2b | M1 | motile |  |  |  | WspR | Pfl01_1058 → | 1227186 | Frame shift | 205* | ∆1bp 557/1002 nt |
| 7.2c | M1 | motile |  |  |  | WspD | Pfl01_1055 → | 1223058 | In-frame ∆ | ∆152-157 | ∆459-476/684 nt |
| 7.2d | M1 | motile |  |  |  | WspD | Pfl01_1055 → | 1223058 | In-frame ∆ | ∆152-157 | ∆459-476/684 nt |
| 7.3a | D1 | non-motile |  |  |  | WspE | Pfl01_1056 → | 1223648 | Missense | M123I | ATG→ATA |
| 7.3b | D1 | non-motile |  |  |  | WspE | Pfl01_1056 → | 1224162 | Missense | R295C | CGC→TGC |
| 7.4a | M2 | motile |  |  |  | WspD | Pfl01_1055 → | 1222912 | Nonsense | Q105* | CAG→TAG |
| 7.4b | M2 | motile |  |  |  | WspE | Pfl01_1056 → | 1224637 | Missense | H453R | CAC→CGC |
| 7.5 | D2 | non-motile |  |  |  | SadC | Pfl01_4451 ← | 5013023 | 5' UTR | 5' UTR | A→C (-11) |
| 7.6 | M3 | motile |  |  |  | SadC | Pfl01_4451 ← | 5012937 | In-frame ∆ | 25L 4→3 | ∆74‑76/1107 nt |
| 7.7 | D3 | non-motile |  |  |  | MorA | Pfl01_4876 → | 5498240 | Missense | S733L | TCG→TTG |
| 8.1 | D0 | non-motile |  |  |  | WspF | Pfl01_1057 → | 1226036 | Missense | I156R | ATC→CGA |
| 8.2a | M1 | motile |  |  |  | WspC | Pfl01_1054 → | 1222439 | Nonsense | Q372* | CAG→TAG |
| 8.2b | M1 | motile |  |  |  | WspD | Pfl01_1055 → | 1223283 | Missense | *228C (WspD::E) | TGA→TGC |
| 8.3a | D1 | non-motile |  |  |  | GcbC | Pfl01_4665 →/ → Pfl01_4666 | 5266677 | 5' UTR | + Pfl01_2031-2032 | +1316 +54/‑56 |
| 8.3b | D1 | non-motile |  |  |  | MorA | Pfl01_4876 → | 5499015 | Missense | M991I | ATG→ATA |
| 8.3c | D1 | non-motile |  |  |  | MorA | Pfl01_4876 → | 5499286 | Missense | E1082K | GAA→AAA |
| 8.4 | M2 | motile |  |  |  | MorA | Pfl01_4876 → | 5497033 | Frame shift | 487* | +4bp 991/3849nt |
| 8.5a | D2 | non-motile |  |  |  | DgcH | Pfl01_0050 ← | 55773 | In-frame ∆ | ∆413-491 | ∆1237-1473/2091 nt |
| 8.5b | D2 | non-motile |  |  |  | DgcX | Pfl01_3550 → | 4023270 | Missense | M350I | ATG→ATA |
| 8.6 | M3 | motile |  |  |  | DgcX | Pfl01_3550 → | 4023085 | In-frame ∆ | ∆289 | ∆3bp 865-867/1680 nt |
| 8.7a | D3 | non-motile |  |  |  | YfiN | Pfl01_0692 → | 818891 | Missense | P186R | CCG→CGG |
| 8.7b | D3 | non-motile |  |  |  | FliI | Pfl01_1539 → | 1729003 | Frame shift | Frame shift | +1bp 1015/1359 nt |
| 9.1 | D0 | non-motile |  |  |  | WspF | Pfl01_1057 → | 1226393 | Missense | G275W | GGG→TGG |
| 9.2 | M1 | motile |  |  |  | WspE | Pfl01_1056 → | 1223361 | Frame shift | 38* | ∆82-127/2295 nt |
| 9.3 | D1 | non-motile |  |  |  | YfiN | Pfl01_0692 → | 818803 | Missense | G157S | GGT→AGT |
| 9.4 | M2 | motile |  |  |  | YfiN | Pfl01_0692 → | 819523 | Nonsense | Q397* | CAA→TAA |
| 9.5a | D2 | non-motile |  |  |  | DgcH | Pfl01_0050 ← | 55896 | In-frame ∆ | ∆445-450 | ∆1333‑1350/2091 nt |
| 9.5b | D2 | non-motile |  |  |  | DgcH | Pfl01_0050 ← | 55647 | In-frame ∆ | ∆524-537 | ∆1558‑1599/2091 nt |
| 9.6a | M3 | motile |  |  |  | tRNA | Pfl01_R0084 ← | 5451395 | 5' UTR | 5' UTR | G→T 49/77nt |
| 9.6b | M3 | motile |  |  |  | RplV | Pfl01_5074 ← | 5702436 | Missense | A43T | GCC→ACC |
| 10.1 | D0 | non-motile | 301.71 | 271.29 | 205.50 | DgcH | Pfl01_0050 ← | 55835 | In-frame ∆ | ∆467-470 | ∆1400‑1411/2091 nt |
| 10.2 | M1 | motile | 48.63 | 48.06 | 46.64 | CalM | Pfl01_1895 → | 2166348 | Frame shift | Frame shift | +5bp 1685/2652 nt |
| 10.3a | D1 | non-motile |  |  |  | CalM | Pfl01_1895 → | 2166348 | In-frame ∆ | In-frame ∆ | -5bp 1685/2652 nt |
| 10.3b | D1 | non-motile |  |  |  | WspF | Pfl01_1057 → | 1226204 | Nonsense | E212* | GAA→TAA |
| 10.3c | D1 | non-motile |  |  |  | CalM | Pfl01_1895 → | 2166348 | In-frame ∆ | In-frame ∆ | -5bp 1685/2652 nt |
| 10.4 | M2 | motile |  |  |  | WspR | Pfl01_1058 → | 1226823 | Missense | L65Q | CTG→CAG |
| 10.5 | D2 | non-motile |  |  |  | CalM | Pfl01_1895 → | 2166348 | In-frame ∆ | In-frame ∆ | -5bp 1685/2652 nt |
| 11.1 | D0 | non-motile | 80.66 | 94.75 | 112.01 | WspA | Pfl01_1052 → | 1220312 | Missense | A381V | GCC→GTC |
| 11.2 | M1 | motile | 49.17 | 51.38 | 65.08 | RndA | Pfl01_2749 ← | 3170622 | Nonsense | Q51* | CAG→TAG |
| 11.3 | D1 | non-motile | 147.33 | 132.67 | 152.43 | DgcH | Pfl01_0050 ← | 55647 | In-frame ∆ | ∆524-537 | ∆1558‑1599/2091 nt |
| 11.4 | M2 | motile | 43.58 | 37.94 | 43.96 | WspR | Pfl01_1058 → | 1226967 | Missense | A113D | GCC→GAC |
| 12.1 | D0 | non-motile |  |  |  | WspE | Pfl01_1056 → | 1225222 | Missense | D648G | GAC→GGC |
| 12.2 | M1 | motile |  |  |  | WspC | Pfl01_1054 → | 1222526 | Frame shift | 421* | ∆1201-1202/1278 nt |
| 12.3 | D1 | non-motile |  |  |  | WspA | Pfl01_1052 → | 1220020 | In-frame ∆ | ∆283-310 | ∆850‑933/1623 nt |
| 13.1 | D0 | non-motile |  |  |  | WspF | Pfl01_1057 → | 1225581 | Missense | A4E | GCA→GAA |
| 13.2 | M1 | motile |  |  |  | WspC | Pfl01_1054 → | 1222479 | Missense | H385P | CAC→CCC |
| 14.1 | D0 | non-motile |  |  |  | WspF | Pfl01_1057 → | 1226127 | Missense | H186P | CAT→CCT |
| 14.2 | M1 | motile |  |  |  | WspA | Pfl01_1052 → | 1220333 | Missense | S388F | TCC→TTC |
| 15.1 | D0 | non-motile |  |  |  | WspE | Pfl01_1056 → | 1225307 | Duplication | 673GMD 1→2 | +9bp 2028/2295 nt |
| 15.2 | M1 | motile |  |  |  | WspF | Pfl01_1057 → | 1226474 | Missense | S302R | AGT→CGT |
| 16.1 | D0 | non-motile |  |  |  | WspF | Pfl01_1057 → | 1225883 | Missense | P105S | CCG→TCG |
| 16.2 | M1 | motile |  |  |  | WspC | Pfl01_1054 → | 1221551 | Nonsense | E76* | GAA→TAA |
| 17.1 | D0 | non-motile |  |  |  | WspF | Pfl01_1057 → | 1226391 | Missense | T274I | ACA→ATA |
| 17.2 | M1 | motile |  |  |  | WspA | Pfl01_1052 → | 1219453 | Nonsense | Q95* | CAG→TAG |
| 18.1 | D0 | non-motile |  |  |  | WspF | Pfl01_1057 → | 1225721 | In-frame ∆ | ∆48-52 | ∆151-165/1011 nt |
| 18.2 | M1 | motile |  |  |  | WspR | Pfl01_1058 → | 1226688 | Nonsense | L20* | TTG→TAG |
| 19.1 | D0 | non-motile |  |  |  | WspA | Pfl01_1052 → | 1220020 | In-frame ∆ | ∆283-310 | ∆850‑933/1623 nt |
| 19.2 | M1 | motile |  |  |  | WspE | Pfl01_1056 → | 1223907 | Frame shift | Frame shift | -1 bp 628/2295 |
| 20 | D0 | non-motile |  |  |  | WspF | Pfl01_1057 → | 1226559 | Nonsense | L330* | TTG→TAG |
| 21 | D0 | non-motile |  |  |  | WspF | Pfl01_1057 → | 1225721 | In-frame ∆ | ∆48-52 | ∆151-165/1011 nt |
| 22 | D0 | non-motile |  |  |  | WspA | Pfl01_1052 → | 1220020 | In-frame ∆ | ∆283-310 | ∆850‑933/1623 nt |
| 23 | D0 | non-motile |  |  |  | WspF | Pfl01_1057 → | 1225883 | Missense | P105S | CCG→TCG |
| 24 | D0 | non-motile |  |  |  | MorA | Pfl01_4876 → | 5499286 | Missense | E1082K | GAA→AAA |
| 25 | D0 | non-motile |  |  |  | WspE | Pfl01_1056 → | 1224324 | Missense | R349S | CGC→AGC |
| 26 | D0 | non-motile |  |  |  | MorA | Pfl01_4876 → | 5498817 | Missense | M925I | ATG→ATA |
| 27.0 ** | M0 | motile |  |  |  | WspC | Pfl01_1054 → | 1221330 | In-frame ∆ | ∆*wspCD* | Δ1954/1954 nt |
| 27.1 | D0 | non-motile |  |  |  | DgcH | Pfl01_0050 ← | 55873 | Missense | Q458P | CAG→CCG |
| 27.2 | M1 | motile | 42.70 | 39.96 | 36.29 | DgcH | Pfl01_0050 ← | 56803 | Missense | Q148P | CAG→CCG |
| 27.3a | D1 | non-motile | 47.26 | 71.96 | 72.99 | IlvH | Pfl01_4787 ← | 5402983 | Missense | A36E | GCG→GAG |
| 27.3b | D1 | non-motile |  |  |  | FlgL | Pfl01_1507 ← | 1687852 | Frame shift | 326* | ∆1bp 866/1572 nt |
| 27.4 | M2 | motile |  |  |  | WspB | Pfl01_1053 → | 1221329 | Missense | *179C (WspB+) | TGA→TGC |
| 27.5 | D2 | non-motile |  |  |  | MorA | Pfl01_4876 → | 5498222 | Missense | V727A | GTA→GCA |
| *ΔrsmE* * | M0 | motile | 61.11 | 61.63 | 60.13 | RsmE | Pfl01_1912 ← | 2185977 | In-frame ∆ | ∆*rsmE* | ∆1‑195/195 nt |
| 28.1 | D0 | non-motile |  |  |  | WspA | Pfl01_1052 → | 1220020 | In-frame ∆ | ∆283-310 | ∆850‑933/1623 nt |
| 28.2 | M1 | motile |  |  |  | WspA | Pfl01_1052 → | 1220716 | Nonsense | Q516* | CAG→TAG |
| 29.1 | D0 | non-motile |  |  |  | WspE | Pfl01_1056 → | 1225506 | Missense | K743Q | AAG→CAG |
| 29.2a | M1 | motile |  |  |  | WspC | Pfl01_1054 → | 1221416 | Frame shift | 51* | + 19bp 91/1278 nt |
| 29.2b | M1 | motile |  |  |  | WspA | Pfl01_1052 → | 1219453 | Nonsense | Q95* | CAG→TAG |
| 30.1 | D0 | non-motile |  |  |  | WspF | Pfl01_1057 → | 1226123 | Nonsense | Q185* | CAG→TAG |
| 30.2a | M1 | motile |  |  |  | WspR | Pfl01_1058 → | 1227012 | Frame shift | 138* | ∆1bp 383/1002 nt |
| 30.2b | M1 | motile |  |  |  | WspC | Pfl01_1054 → | 1222448 | Frame shift | 389* | ∆1bp 1123/1278 nt |
| 31.1 | D0 | non-motile |  |  |  | WspC | Pfl01_1054 → | 1222342 | In-frame ∆ | WspC::D | ∆305bp WspC:WspD |
| 31.2a | M1 | motile |  |  |  | WspA | Pfl01_1052 → | 1220714 | Missense | R515P | CGT→CCT |
| 31.2b | M1 | motile |  |  |  | WspA | Pfl01_1052 → | 1220099 | Missense | T310N | ACC→AAC |
| 32.1 | D0 | non-motile |  |  |  | WspE | Pfl01_1056 → | 1225223 | Missense | D648G | GAC→GAA |
| 32.2a | M1 | motile |  |  |  | WspA | Pfl01_1052 → | 1219957 | Missense | S263P | TCC→CCC |
| 32.2b | M1 | motile |  |  |  | WspB | Pfl01_1053 → | 1221235 | Nonsense | W148* | TGG→TAG |
| 33.1 | D0 | non-motile | 86.13 | 60.82 | 85.85 | WspF | Pfl01_1057 → | 1225721 | In-frame ∆ | ∆48-52 | ∆151-165/1011 nt |
| § Relationships of the lineages presented here are visualized in Fig. S1. Isolate IDs correspond to variant calling files found in ‘Table S1 - Source Data 1’ that provides evidence for each reported mutation. All isolates were generated in this experiment unless otherwise noted or stated here: * (Kim et al., 2014); ** (Kim et al., 2016); # (Compeau et al., 1988). M and D served as the reference ancestral isolates throughout this study.  §§ Generation denotes sequential phenotype transitions within each lineage: WT, M0 (M isolate from WT), D0 (D isolate from M0), M1 (second M isolate that evolved from , D1, … M6, D6.  † Determined by stab-motility assays.  ‡ Select isolates were chosen for LC-MS/MS c-di-GMP quantification conducted in triplicate.  *** Locus, CDS, and Mutation Location (NT) were extracted from GenBank reference CP000094 unless otherwise stated. CDS: → (+ strand), ← (- strand), / (intergenic space). Genotype (AA/NT) was reported by BreSeq during variant analysis. Consequence describes the nature of mutation. | | | | | | | | | | | |
