# Supplementary material for: Identification of Cyclic-di-GMP-Modulating Protein Residues by Bidirectionally Evolving a Social Behavior in Pseudomonas fluorescens
Source: mSystems. 2022 Oct 3;7(5):e00737-22. doi: 10.1128/msystems.00737-22 (PMC9600634; doi:10.1128/msystems.00737-22)
Supplement: TABLE S2 [file msystems.00737-22-s0005.docx]

| **Supplemental Table S2.** | | | | | | | | | | |
| --- | --- | --- | --- | --- | --- | --- | --- | --- | --- | --- |
| Source | Target | Attribute | Count * |  |  |  | Source | Target | Attribute | Count |
| CdrB | DgcH | D | 1 |  |  |  | WspA | WspD | M | 3 |
| CdrB | MorA | D | 1 |  |  |  | WspA | WspE | M | 5 |
| ParA | WspA | D | 1 |  |  |  | WspB | MorA | D | 1 |
| ParA | WspB | D | 1 |  |  |  | WspB | WspE | D | 2 |
| CalM | CalM | D | 2 |  |  |  | WspB | IlvI | M | 2 |
| CalM | WspF | D | 1 |  |  |  | WspC | DgcH | D | 1 |
| RndA | DgcH | D | 1 |  |  |  | WspC | WspA | D | 2 |
| NarA | WspD | M | 1 |  |  |  | WspC | WspE | D | 1 |
| DgcY | Pfl01_1567 | D | 1 |  |  |  | WspC | WspD | M | 2 |
| DgcY | FlgF | D | 1 |  |  |  | WspC | WspE | M | 1 |
| DgcY | DgcY | M | 1 |  |  |  | WspC | WspR | M | 1 |
| DgcX | Pfl01_1539 | D | 1 |  |  |  | WspD | Pfl01_1562 | D | 1 |
| DgcX | YfiN | D | 1 |  |  |  | WspD | Pfl01_1757 | D | 1 |
| DgcX | DgcX | M | 1 |  |  |  | WspD | SadC | D | 3 |
| SadC | DgcH | D | 2 |  |  |  | WspD | GcbC | D | 2 |
| SadC | IlvH | D | 1 |  |  |  | WspD | MorA | D | 2 |
| SadC | MorA | D | 1 |  |  |  | WspD | WspA | D | 1 |
| SadC | WspE | D | 2 |  |  |  | WspD | WspD | D | 1 |
| SadC | YfiN | D | 2 |  |  |  | WspD | WspE | D | 3 |
| SadC | YfiR | D | 1 |  |  |  | WspD | WspA | M | 3 |
| SadC | SadC | M | 4 |  |  |  | WspE | Pfl01_3026 | D | 1 |
| DgcH | FlgL | D | 1 |  |  |  | WspE | DgcY | D | 1 |
| DgcH | IlvH | D | 1 |  |  |  | WspE | SadC | D | 2 |
| DgcH | WspA | D | 2 |  |  |  | WspE | DgcH | D | 2 |
| DgcH | WspF | D | 1 |  |  |  | WspE | GcbC | D | 1 |
| DgcH | CalM | M | 1 |  |  |  | WspE | MorA | D | 2 |
| DgcH | DgcH | M | 2 |  |  |  | WspE | WspA | D | 2 |
| DgcH | R0084 | M | 1 |  |  |  | WspE | WspE | D | 1 |
| DgcH | RplV | M | 1 |  |  |  | WspE | YfiN | D | 1 |
| DgcH | WspR | M | 1 |  |  |  | WspE | WspA | M | 2 |
| GcbC | DgcY | D | 1 |  |  |  | WspE | WspB | M | 1 |
| GcbC | GcbC | D | 1 |  |  |  | WspE | WspC | M | 2 |
| GcbC | MorA | D | 2 |  |  |  | WspE | WspE | M | 2 |
| GcbC | GcbC | M | 1 |  |  |  | WspE | WspR | M | 2 |
| IlvH | ParA | M | 1 |  |  |  | WspF | WspA | M | 4 |
| IlvH | WspB | M | 1 |  |  |  | WspF | WspC | M | 4 |
| IlvI | DgcH | D | 1 |  |  |  | WspF | WspD | M | 1 |
| IlvI | WspE | D | 3 |  |  |  | WspF | WspE | M | 2 |
| MorA | DgcX | D | 1 |  |  |  | WspF | WspR | M | 4 |
| MorA | DgcH | D | 1 |  |  |  | WspR | CalM | D | 2 |
| MorA | MorA | M | 1 |  |  |  | WspR | GcbC | D | 1 |
| RsmE | DgcH | D | 2 |  |  |  | WspR | MorA | D | 1 |
| RsmE | WspA | D | 4 |  |  |  | WspR | YfiN | D | 1 |
| RsmE | WspD | D | 1 |  |  |  | YfiN | DgcX | D | 1 |
| RsmE | WspE | D | 2 |  |  |  | YfiN | DgcH | D | 3 |
| RsmE | WspF | D | 15 |  |  |  | YfiN | GcbC | D | 2 |
| RsmE | WspD | M | 1 |  |  |  | YfiN | IlvH | D | 1 |
| WspA | NarA | D | 1 |  |  |  | YfiN | MorA | D | 3 |
| WspA | DgcH | D | 1 |  |  |  | YfiN | RapA | D | 1 |
| WspA | WspA | D | 3 |  |  |  | YfiN | WspE | D | 2 |
| WspA | WspE | D | 2 |  |  |  | YfiN | CdrB | M | 1 |
| WspA | RndA | M | 1 |  |  |  | YfiN | YfiN | M | 4 |
| WspA | WspA | M | 4 |  |  |  | YfiR | YfiN | M | 1 |
| WspA | WspB | M | 1 |  |  |  |  |  |  |  |
| * Values are reported in to-from format with source indicating the mutation a parent isolate carries, target indicating the mutation a daughter cell carries, attribute indicating if said daughter cell’s mutation resulted in the M or D phenotype, and count indicating the frequency of the pattern in the dataset. | | | | | | | | | | |
