# Supplementary material for: Identification of Cyclic-di-GMP-Modulating Protein Residues by Bidirectionally Evolving a Social Behavior in Pseudomonas fluorescens
Source: mSystems. 2022 Oct 3;7(5):e00737-22. doi: 10.1128/msystems.00737-22 (PMC9600634; doi:10.1128/msystems.00737-22)
Supplement: TABLE S3 [file msystems.00737-22-s0006.docx]

| **Supplemental Table S3.** | | |  |
| --- | --- | --- | --- |
| Target mutation | Forward Primer Sequence | Reverse Primer Sequence | |
|  |  |  |  |
| WspA::D | ATCTGGTGAACGCCAAACT | TGACCAGGCAATCTTCCATC | |
| WspB::D | CCACGTATGACATCGAACAGA | CTGTCGAGATCGAGCAGTTC | |
| WspC::D | TGCCGAGAACGAAGGTTA | CTGTCGAGATCGAGCAGTTC | |
| IlvH (A36E) | CAAGACGGAGCGTACCTAATC | AACGGTAGCCGAACCTTTAC | |
| IlvH (S149R) | CAAGACGGAGCGTACCTAATC | AACGGTAGCCGAACCTTTAC | |
| IlvH (G150S) | CAAGACGGAGCGTACCTAATC | AACGGTAGCCGAACCTTTAC | |
| IlvI (Δ17bp) | AGGGAAGCTCAAAGCGATAC | AACCTGCCGGTTACCAATAC | |
| IlvI (Δ 23bp) | AGGGAAGCTCAAAGCGATAC | AACCTGCCGGTTACCAATAC | |
| DgcY (Δ3505-3506) | ATGGCTAAGCCCAGTTACACA | TGAAGCCGTTGAGATCGATA | |
| DgcY (Δ3500-3510) | ATCGTTAGGGCGCTATCAATAC | TTGCATCGAGCAGGGTTTTC | |
| NarA (A383S) | CATTTACACCTCGGGAGATGAC | CGACAGCGCATTGAAGAAAC | |
| CalM (+5bp) | CTGCTGACCAATCCAATCCT | AAGTCATCGTCTGCCAACTC | |
| RndA (Q51S) | TTGTTCACTCATCGCCTACTG | CGACCAGCGTTCCGATAAA | |
| CdrB (A290T) | CGTTATCAACAGGACGGCTATC | GCTTGAGTTCCAGACCGTTATT | |
| *ilvH* external check | CGCACTCCTACATGGAATCA | CAGCTACATCGGTCACTTTCA | |
| *ilvI* external check | AAGCCAAACTGCGTTCCTA | TTCATCTGGATACTGCCTTCAC | |
